# Enrichment of genetic markers of recent human evolution in educational and cognitive traits

**DOI:** 10.1038/s41598-018-30387-9

**Published:** 2018-08-22

**Authors:** Saurabh Srinivasan, Francesco Bettella, Oleksandr Frei, W. David Hill, Yunpeng Wang, Aree Witoelar, Andrew J. Schork, Wesley K. Thompson, Gail Davies, Rahul S. Desikan, Ian J. Deary, Ingrid Melle, Torill Ueland, Anders M. Dale, Srdjan Djurovic, Olav B. Smeland, Ole A. Andreassen

**Affiliations:** 1 NORMENT, KG Jebsen Centre for Psychosis Research, Institute of Clinical Medicine, University of Oslo, Oslo, Norway; 2 Division of Mental Health and Addiction, Oslo University Hospital, Oslo, Norway; 3 Centre for Cognitive Ageing and Cognitive Epidemiology, University of Edinburgh, Edinburgh, UK; 4 Department of Psychology, University of Edinburgh, Edinburgh, UK; 5 Multimodal Imaging Laboratory, University of California at San Diego, La Jolla, CA USA; 6 Center for Human Development, University of California at San Diego, La Jolla, CA USA; 7 Institute of Biological Psychiatry, Mental Health Center St. Hans, Mental Health Services Copenhagen, Roskilde, Denmark; 8 Department of Family Medicine and Public Health, University of California, San Diego, La Jolla, CA USA; 9 Neuroradiology Section, Department of Radiology and Biomedical Imaging, University of California at San Francisco, San Francisco, CA USA; 10 Department of Psychiatry, University of California, San Diego, La Jolla, CA USA; 11 Department of Medical Genetics, Oslo University Hospital, Oslo, Norway; 12 NORMENT, KG Jebsen Centre for Psychosis Research, Department of Clinical Science, University of Bergen, Bergen, Norway

## Abstract

Higher cognitive functions are regarded as one of the main distinctive traits of humans. Evidence for the cognitive evolution of human beings is mainly based on fossil records of an expanding cranium and an increasing complexity of material culture artefacts. However, the molecular genetic factors involved in the evolution are still relatively unexplored. Here, we investigated whether genomic regions that underwent positive selection in humans after divergence from Neanderthals are enriched for genetic association with phenotypes related to cognitive functions. We used genome wide association data from a study of college completion (N = 111,114), one of educational attainment (N = 293,623) and two different studies of general cognitive ability (N = 269,867 and 53,949). We found nominally significant polygenic enrichment of associations with college completion (p = 0.025), educational attainment (p = 0.043) and general cognitive ability (p = 0.015 and 0.025, respectively), suggesting that variants influencing these phenotypes are more prevalent in evolutionarily salient regions. The enrichment remained significant after controlling for other known genetic enrichment factors, and for affiliation to genes highly expressed in the brain. These findings support the notion that phenotypes related to higher order cognitive skills typical of humans have a recent genetic component that originated after the separation of the human and Neanderthal lineages.

## Introduction

The evolution of cognitive function and brain development is regarded as the result of a complex interplay of nature and nurture, where development seems to be driven by genes and shaped by environment^1^. Modern humans have highly complex brains, capable of processing vast information and solving abstract problems. In addition, humans have enhanced cognitive functioning, especially in the domains of cooperation, egalitarianism, theory of mind, language and culture, and achieved new modes of thinking and reasoning that seem to have greatly increased their ability to flourish as a species^2^. Archaeological and fossil records provide evidence suggesting anatomical and morphological evolution of humans and their predecessors^3,4^. However, we have no direct evidence for the evolution of higher cognitive functions and must rely on cultural artefacts that indirectly suggest behavioural changes^5^.

Several lines of evidence support the idea that humans have developed complex language^5^, executive functioning, and abstract thinking in the process of evolution^6^. Language, and the thoughts that it expresses, arguably constitute the most distinctive features of the modern human mind^7^. With specialized knowledge and means to communicate, humans seem to be able to create efficient tools, codify knowledge, make rules and organize society^8^. While we do not know how Neanderthals would have performed in cognitive tests, cognitive studies comparing humans and chimpanzees have found different patterns of performance across cognitive domains suggesting a role of evolution in specific higher cognitive functions^9^. Anatomically, we observe increased general cephalization, which is said to be associated with greater behavioural complexity, and enlargement and specialization of brain regions adapting to various sensory uses over the course of evolution^10^. Language and social skills are said to have evolved together; better language skills would be required to facilitate better cooperation while hunting and foraging as a group. Meanwhile, living in social groups may have helped in the development of the neocortex, or the human “social brain”^11^.

Evolutionary psychologists discuss whether our cognitive processes and environment co-evolved incrementally, instead of undergoing a dramatic change^1,12^. Recent studies suggest that Neanderthals could have had some ability to express themselves artistically and some sort of proto culture or religion^13^. This is in line with the notion that human intelligence, while not completely innate, is shaped by natural selection and evolutionary processes which helped the species adapt to the environment. Genes and cultures are suggested to co-evolve^14^, as in the case of the human ability to learn as a group, which seems to set them apart from our primate relatives and shaped human specific phenotypes. Social learning could help humans to acquire new skills faster and to enhance them^15^. Cognitive function and academic achievement, while influenced by genes, probably need the right environment to achieve full potential^16^. Educational attainment (or the ability to complete college) is governed not only by many socio-economic, political and cultural factors^17,18^ but also by genetic factors that were estimated to account for approximately 20% of the phenotypic variance^19^. In twin studies, the heritability of general cognitive function was estimated to be 50–60%^20,21^. Educational attainment is also related to cognitive performance with a high genetic correlation^22,23^. Cognitive function is in turn often associated with neuropsychiatric phenotypes, with which it shows some genetic overlap^16,24–27^. Finally, several cognitive traits, such as attention, memory and reasoning, are important to succeed in academics.

Neanderthals, considered a sister group of modern humans, are suggested to have split from the human lineage between 500,000 to 750,000 years ago^28–30^. As the closest living relatives to humans, chimpanzees are often used as a reference point for ancestral alleles. Chimpanzees split from humans approximately 6.3 million years ago^31^. The chimpanzee genome was sequenced in 2005, and aligns with 96 to 98% of the human genome^32,33^ depending on the exact criterion utilized for sequence alignment. Recent studies have found that variants associated with cognitive function are enriched in regions of the genome that are evolutionarily conserved in mammals^34^. However, these do not provide a human specific time frame. The availability of Neanderthal and chimpanzee genomes makes it possible to determine when in the course of human evolution genomic regions underwent selective pressures by cross-referencing the chimpanzee, human and Neanderthal sequences. This provides a rare opportunity to gain novel insight into the evolutionary processes in humans.

We have previously shown how these available genomes can be used to detail the evolution of schizophrenia, employing the original version^29^ of the Neanderthal selective sweep score^35^. Here, we applied the same polygenic enrichment approach in conjunction with a more recent and comprehensive post-Neanderthal selective sweep index^36^ to study the evolutionary aspects of educational attainment and cognitive function, and determine enrichment in genomic regions that may have undergone recent positive selection in humans. To this end, we analysed genome-wide association (GWAS) summary statistics for two measures of educational attainment, college completion (College)^37^ and years of educational attainment (EduYears)^38^, and two measures of fluid intelligence from two studies of general cognitive ability (GCA)^39,40^. The GCA metric is designed to capture around 40–50% of the variance across diverse cognitive abilities, irrespective of the specific tests used to construct it^41,42^. We hypothesized that higher cognitive functions are a product of human evolution in line with previous theories^43^. We compared the cognitive phenotypes to height and body mass index (BMI), two human traits with GWASs of similar size, to assess the specificity of this enrichment.

## Results

**Figure 1:**
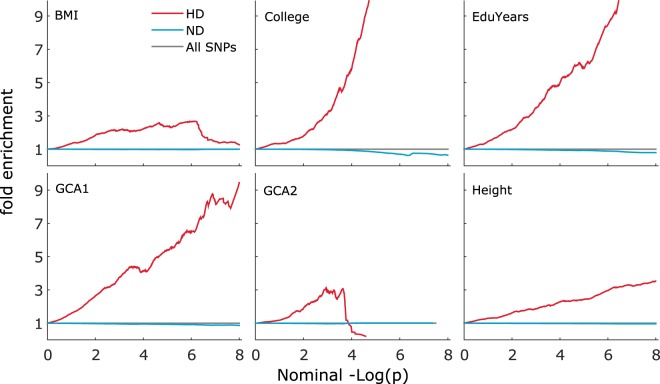
Fold enrichment plots of phenotypes stratified according to Neanderthal selective sweep score. Shown are the fold enrichment plots of GWAS summary statistics *p*-values for body mass index (BMI), college completion (College), educational attainment (EduYears), general cognitive ability (GCA1 and 2) and height stratified based on Neanderthal selective sweep (NSS) score. The human divergent (HD) stratum comprises single nucleotide polymorphisms (SNPs) with negative NSS scores. The non-divergent (ND) stratum comprises all SNPs with positive NSS scores. HD SNPs show some upward deflection from ND and all SNPs. This signifies a comparatively higher proportion of low p-values among HD SNPs.

Utilizing a post-Neanderthal selective sweep (PNSS) index^36^, we assessed the effect of a variant’s affiliation to the selectively swept regions of the genome on traits related to cognition: College completion (College) (N = 111,114), education attainment (EduYears) (N = 293,623) and two measures of general cognitive ability (GCA), GCA1 (N = 269,867) and GCA2 (N = 53,949)). The PNSS index defines regions that have undergone positive selection in humans after the separation of the human and Neanderthal lineages. We specifically investigated association enrichment, visualized as an upward deflection in fold enrichment plots and a leftward deflection in Q-Q plots (for details, see Methods). The fold enrichment plots (Fig. 1**)** and conditional Q-Q plots **(**Supplementary Fig. S1) suggest that the genetic variants in regions that may have undergone positive selection in humans, i.e. the human divergent (HD) regions, are markedly enriched of associations with College, EduYears and GCA1, and to a lesser extent with GCA2.

To test the significance of the enrichment, we conducted a stratified LD score regression analysis^44^. The LD score regression method provides an estimate of the fold enrichment associated with these evolutionary regions, and thus an estimate of enrichment^45^. Specifically, we found significant enrichment for EduYears (fold enrichment = 2.00 vs. expected, p = 0.044), GCA2 (3.48, p = 0.023), College (2.33, p = 0.026) and GCA1 (2.12, p = 0.024) (Table 1). The LD score regression analysis also provides a regression coefficient controlling for affiliation to generic functional categories such as intron, exon, 3′UTR, 5′UTR, and to brain genes in particular (Supplementary Tables S1-S2**)**. Our analysis indicates that affiliation to HD regions significantly contributes to the LD score effect in College (p = 0.019), GCA2 (p = 0.020), GCA1 (p = 0.029) and EduYears (p = 0.046) after controlling for the other covariates.

**Table 1:** Post-Neanderthal selective sweep enrichment.

Phenotype	Enrichment	
	Fold ± s.e.m	p-value
BMI	1.38 ± 0.374	3.12 × 10^−1^
College	2.33 ± 0.605	2.63 × 10^−2*^
EduYears	2.00 ± 0.605	4.48 × 10^−2*^
GCA 1	2.15 ± 0.579	2.45 × 10^−2*^
GCA 2	3.48 ± 1.099	2.36 × 10^−2*^
Height	2.10 ± 0.607	7.17 × 10^−2^

^*^Nominally significant.

Stratified enrichment analysis for genetic variants in regions that possibly underwent a selective sweep after divergence from Neanderthal. Cognitive measures: college completion (College), educational attainment (EduYears), general cognitive ability (GCA1 and 2); Anthropometric measures: body mass index (BMI) and Height. The table shows the LD score regression model enrichment test statistics: the enrichment of associations among variants in swept regions compared to other variants (Fold), and the corresponding p-values.

To further detail the specificity of the enrichment, we used summary statistics from height and BMI GWASs, which involved sample sizes comparable to or larger than the ones available to the cognitive GWASs. While height and BMI have been associated with some evolutionary pressure in humans^46,47^, we expect them to show a less pronounced enrichment than the cognitive phenotypes since they are less likely to be human specific compared to cognitive function.

Our fold enrichment plots (Fig. 1) suggest the presence of some enrichment in Height and BMI among variants in HD regions. The deflections are more consistent than observed for GCA2 but less pronounced than seen for College, EduYears and GCA1. The fold enrichment test statistics (BMI: 1.38, p = 0.312; Height: 2.10, p = 0.072) are non-significant, as are the regression coefficients (BMI p = 0.252 and Height p = 0.080). Upon meta-analysing the stratified LD-score enrichment test statistics for anthropometric and cognitive traits, a significant effect is found for the latter (Fisher-combined test p = 4.00 × 10^−4^) but not for the former (Fisher-combined test p = 0.107).

**Figure 2:**
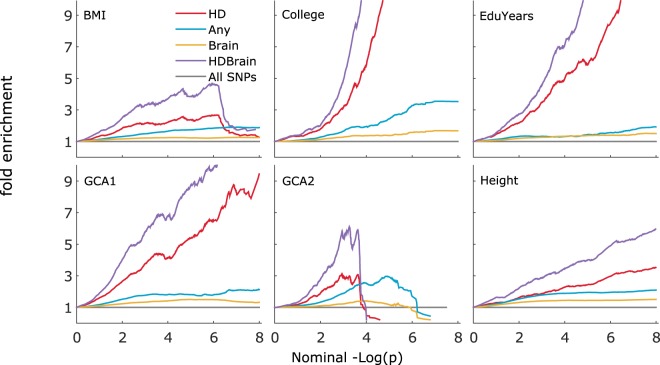
Enrichment of associations with various traits stratified according to their affiliation to Post-Neanderthal selective sweep regions and brain genes. Fold enrichment plots for body mass index (BMI), college completion (College), educational attainment (EduYears), general cognitive ability (GCA1 and 2) and height. Plots are shown for variants annotated to human divergent regions (HD), variants annotated to generic genes (Any), variants annotated to genes with high expression in the brain (Brain), variants in HD regions annotated to genes with high expression in brain (HDBrain), and all variants (All SNPs). The HDBrain category is enriched (upward deflection) compared with the other categories (i.e., presents a higher incidence of associations [lower p -values]).

Given the importance of genes involved in brain function for the phenotypes of interest, and their evolutionary relevance, we performed additional analyses targeting genes with high expression levels in the brain (brain genes) (Fig. 2). Brain genes in HD regions show more pronounced association enrichment than any brain genes or any SNPs in HD regions in the fold enrichment plots. The stratified LD-score regression analysis suggests brain genes in the HD regions (HD Brain) to be more enriched than any SNPs in HD regions (HD) in GCA2 (4.75 vs. 3.48) but not in College, EduYears and GCA1 (College = 2.16 vs. 2.33, EduYears = 1.83 vs. 2.00, GCA1 = 1.68 vs. 2.12). However, the numbers of SNPs in these strata are very low and the stratified LD-score regression analysis does not give conclusive results (Supplementary Table S2).

## Discussion

Applying our Neanderthal polygenic enrichment approach to recent large GWAS data on cognitive traits^37–39,48^, we investigated the hypothesis that higher cognitive functions in humans have a recent evolutionary component. We assessed the extent to which the cognitive phenotypes, College, EduYears, and GCA are affected by genetic variation in regions of the human genome that may have undergone selective sweeps since divergence from the Neanderthals and found nominally significant enrichment with all cognitive traits. Together, these findings lend support to the hypothesis that higher cognitive traits typical of humans have a component that originated after the separation of the human and Neanderthal lineages, in line with previous theories^43^.

The fold enrichment and Q-Q plots for the three cognitive phenotypes showed various degrees of deflection in the HD stratum (Fig. 1 and Supplementary Fig. S1). The fold enrichment statistics confirmed consistently significant enrichments for College, EduYears, and two independent data sets on GCA. The phenotypes summarized under GCA measure fluid intelligence and capture the shared variance across cognitive traits, irrespective of the tests applied. GCA is also phenotypically and genetically correlated with educational attainment^37,49^.

The meta-analysis of the stratified LD-score enrichment tests statistics suggests that the enrichments detected here are somewhat specific to cognitive phenotypes. In a previous study, we found Height and BMI to be significantly enriched in HD regions. However, those results were obtained using the original Neanderthal selective sweep score^29^ and a different statistical method^35^.

The current study sheds light on the evolutionary architecture of human cognitive traits. These phenotypes are complex and involve many genetic and environmental factors. Our results suggest an underlying evolutionary factor in the genetics of educational attainment and cognitive function. Recent studies have indeed found that genes associated with educational attainment are under selective pressure^50^. Behavioural studies suggest that humans have evolved to outperform other primates in certain higher cognitive abilities, such as learning through imitation and from the mistakes of others, and are also quicker to learn unfamiliar tasks^51–55^. Paleontological or archaeological findings indicate the increasing complexity of cranial anatomy and material culture during human evolution^3,4^. Our results constitute further evidence that these traits are related to the evolution of modern humans since the divergence from Neanderthals about 500,000 to 750,000 years ago.

The PNSS region affiliation score is an index of possible positive selection in regions of the human genome after divergence from Neanderthals^36^, but provides no direct experimental evidence that positive selection occurred at those sites. Nonetheless, the Neanderthal selective sweep score compiled on the first edition of the Neanderthal sequence by Green^29^, and the subsequent post Neanderthal swept regions identified by Prufer^36^, proved to be more sensitive than other genomic proxies of positive selection like human accelerated regions and segmental duplications used in our previous studies^56^. The Neanderthal sweep scores are also more specific than other measures of negative selection in mammals^34^ in detecting association enrichment.

In the evolution of complex traits, polygenic selection involving subtle shifts of allele frequencies at many loci simultaneously may have been more common than major shifts induced by strong forces^57^. Thus, selection acting simultaneously on many standing variants could have been the more efficient mechanism for phenotypic adaptation^58,59^. The PNSS may therefore not be in the best position to account for genetic drift or other neutral selection forces. However, the distinction between weak and strong selection is not clear. The methods applied in our analyses have been useful in studying several aspects related to polygenic factors in complex human phenotypes before^35,56,60–63^. Although the evolutionary proxy, the PNSS, is designed to detect positive selection sweeps rather than polygenic adaptation, the present results are in line with previous theories positing that higher cognitive functions are a product of human evolution^43^.

While the combined analysis of data from the large cognitive and educational GWASs and the Neanderthal genome sequence is unique to this study, it entails some limitations. The power of the different GWASs depends on their size and on the genetic architecture and polygenicity^64^ of the traits analysed. Hence, the differences in detected enrichments are not strictly comparable to one another. The evolutionary enrichment observed for cognitive phenotypes could be influenced by affiliation to brain genes and genetic functional elements such as introns, exons, 5′UTR and 3′UTR. Despite control for these factors in the analyses, they may be overrepresented in the information-rich portions of the Neanderthal DNA that could be reconstructed. Also, while LD was properly accounted for in the statistical analyses, the enrichment in the plots could be influenced by LD-tagging effects. This could be the reason for the discrepancy observed between the visual and the statistical assessments. Given the complexity of cognitive phenotypes, the observed enrichment may be confounded by other factors with a potential evolutionary component. For example, educational attainment is known to be influenced by other pathologies such as ADHD^65^ and genes associated with ADHD may have some evolutionary advantage as well. Future replication of this result is warranted using summary statistics from larger educational attainment GWAS^66^. Our previous experience suggests that increases in sample size tend to enhance polygenic enrichments^35^.

In conclusion, we demonstrate that the genetic architectures of two measures of educational attainment and two versions of GCA are enriched for genomic regions that were likely subjected to selective sweeps since divergence from Neanderthals. This suggests that some genetic components of higher cognitive functions in humans are driven by more recent evolutionary processes. These findings should be confirmed in independent studies that could also identify the specific genetic variants involved, to inform the biological underpinnings of human cognitive function.

## Materials and Methods

### Samples

Educational attainment has a well-documented health-education gradient as well as phenotypic and genetic relation to cognitive functioning^67^, and is influenced by environmental and genetic factors^19,68^. We obtained summary statistics for about ten million single nucleotide polymorphisms (SNPs) from a GWAS of educational attainment (EduYears)^38^ (sample N = 328,917 Caucasian individuals from North America, Western Europe and Australia), as well as from UK Biobank GWASs of college or university degree (College)^37^ (sample N = 111,114) and of general cognitive ability (GCA1)^40^ (sample N = 269,867). The data on GCA1 was based on 269,867 individuals drawn from 14 cohorts, primarily consisting of data from the UK Biobank (sample N = 195,653) and the Cognitive Genomics Consortium (sample N = 35,289). We also obtained summary statistics for the same set of SNPs from a similar GWAS of GCA (i.e., GCA2) in middle and older age by the CHARGE consortium, which included a total of 53,949 individuals^39^. Finally, we used Height (sample N = 183,727) and BMI (sample N = 339,224) GWAS summary statistics from the GIANT consortium study^69,70^ as control sets.

### Analytical Approach

We employed genetic enrichment methods recently developed to uncover more of the genetic architecture of complex traits^60–62,71^. Specifically, we investigated the enrichment of associations concurrent with the evolutionary affiliations in a covariate-modulated statistical framework^61^. We investigated whether variants in evolutionarily salient regions or tagging other variants therein, are more likely associated with measures of education attainment and general cognitive function, as well as with other control phenotypes. The visual displays of enrichment were produced with MATLAB (www.mathworks.com/products/matlab). The enrichment test statistics were computed using the LD-score package^44^.

### Cognitive phenotypes

College completion (College) measures the highest level of educational qualification achieved^37^ while educational attainment (EduYears)^38^ measures the years of completed schooling. These are used as a proxy for intelligence and they show high genetic correlation^48^. The general cognitive ability is not a specific cognitive skill but a measure of various fluid cognitive ability tests. The measures used in GCA1 and 2 were constructed using the first un-rotated component extracted from a principal component analysis of the individual cognitive test scores that measured general fluid cognitive functions^39^. These measures of fluid intelligence correlate highly with general cognitive ability^40,48,72^. The scores used by these two independent phenotypes capture the shared variance across cognitive test batteries measuring fluid cognitive functions, and explain around 40–50% of the variation across cognitive domains. Further details of the tests administered can be found in the original publications^37,39,40^.

### Post-Neanderthal selective sweep regions

The index of the post-Neanderthal selective sweep (PNSS) regions was obtained from the work of Prüfer *et al*.^36^ and is downloadable from, http://cdna.eva.mpg.de/neandertal/. The authors used a hidden Markov model to identify regions in Neanderthals that differ from modern humans^36^. Neanderthal and Denisovan genes were used to identify regions that differed from the representative modern human population in the 1000 genomes project variants. The identified regions were assigned a score based on genetic lengths and a cut off was assigned for regions that were most likely to have undergone positive selection in humans.

We assigned all SNPs a value of 0 or 1 based on whether these fell outside or inside the regions of recent positive selection in humans, respectively.

### Confounding/mediating effects

We controlled for the following factors while assessing the evolutionary enrichment of cognition/education associations:

#### Brain genes

We used the protein atlas (http://www.proteinatlas.org/humanproteome/brain) to select all genes that are expressed specifically in the brain of *Homo sapiens*. We identified a total of 4915 genes by filtering for genes that have high expression levels in brain. The 1000 Genomes Project SNPs were then aligned with the identified genes. The ones overlapping with these genes were assigned a “Brain” value of 1, the rest were assigned a “Brain” value of 0. All SNPs were subsequently assigned LD–informed “Brain” scores (see below).

#### Annotation of genomic regions, LD-based

The SNPs that fall within certain regions of interest may capture only a limited portion of the association signal ascribable to that region. We used an LD-weighted scoring algorithm^35,56,71^ to identify SNPs that tag specific DNA regions even if they are not situated within them. For each SNP, a pairwise correlation coefficient approximation to LD (*r*^2^) was extracted for all 1KGP SNPs within a 1,000,000 base pairs (1 Mb). All *r*^2^ values <0.2 were set to 0 and each SNP was assigned an *r*^2^ value of 1.0 with itself. LD-weighted region annotation scores for all DNA regions of interest were computed as the sum of LD *r*^2^ between the tag SNP and all 1KGP SNPs in those regions. Given SNP *i*, its LD-weighted region annotation score was computed as LDscore_*i*_ = Σ_*j*_ (*δ*_*j*_ *r*_*ij*_^2^), where *r*_*ij*_^2^ is the LD *r*^2^ between SNP *i* and SNP *j* and *δ*_*ij*_ takes values of 1 or 0 depending on whether the 1KGP SNP*j* is within the region of interest or not. LD scores were assigned to exons, introns, 3′UTR and 5′UTR^35,56,71,73^.

#### Intergenic correction

Intergenic SNPs are defined as having LD-weighted annotation scores for exon, intron, 3′UTR and 5′UTR equal to zero and being in LD with no SNPs in the 1KGP reference panel located within 100,000 base pairs of a protein coding gene, within a non-coding RNA, within a transcription factor binding site or within a miRNA binding site^71^. Those singled out in this way are expected to form a collection of non-genic SNPs not belonging to any annotated functional elements and their LD-associated regions within the genome and therefore represent a collection of likely null associations. Intergenic SNPs were used to estimate the inflation of GWAS summary statistics due to cryptic relatedness. We used intergenic SNPs because their relative depletion of associations suggests they provide a set of reliably null SNPs that is less contaminated by polygenic effects. The inflation factor, λ_GC_, was estimated as the median squared z-score of independent sets of intergenic SNPs across one hundred LD-pruning iterations, divided by the expected median of a chi-square distribution with one degree of freedom.

### Conditional quantile-quantile plots

To visualize enrichment, we constructed conditional quantile-quantile (Q-Q) plots where we compared the nominal p-value distribution to the empirical distribution^71^. In the presence of null relationships, the nominal p-values form a straight line on a Q-Q plot when plotted against the empirical distribution. We plotted −log_10_ nominal p-values against −log_10_ empirical p-values for the two SNP strata subdivided by the PNSS score, as well as for all SNPs. Leftward deflections of the observed distribution from the null line reflect increased tail probabilities in the distribution of test statistics (z-scores) and consequently an over-abundance of low p-values compared to that expected under the null hypothesis^71^. Enrichment is present if the line corresponding to the variants of interest has a leftward deflection from the comparison stratum. To assess polygenic effects below the standard GWAS significance threshold, we focused the Q-Q plots on SNPs with nominal −log10(p) < 7.3 (corresponding to p > 5 × 10^−8^).

### Fold enrichment plots

To visually emphasize the association enrichment, we used conditional fold enrichment plots^74^. As for Q-Q plots, the covariate of interest, i.e. the PNSS score, is used to subdivide SNPs into two strata. The plots were obtained by computing the empirical cumulative distribution of −log10(p)-values for SNP association with a given phenotype for all SNPs, and for the two SNPs strata determined by the PNSS score. Then each stratum’s fold enrichment was calculated as the ratio CDF_stratum_/CDF_all_ between the −log10(p) cumulative distribution for that stratum and the −log10(p) cumulative distribution for all SNPs. The nominal −log10(p) values are plotted on the x-axis, the fold enrichment in the y-axis. To assess polygenic effects below the standard GWAS significance threshold, we focused the fold enrichment plots on SNPs with nominal −log10(p) < 7.3 (corresponding to p > 5 × 10^−8^). Enrichment is present if the line corresponding to the SNPs of interest has an upward deflection. The plots should be interpreted with caution when the baseline is determined by fewer than 5–10 data points.

### Stratified enrichment analysis

To quantify the contribution of variants within the PNSS regions we conducted analyses using an approach^45^ based on stratified LD score regression^44^. We first dichotomized the PNSS scores into binary scores. We used the LD-score tool with the “—h2” option to estimate SNP-based heritability of variants with negative NSS score, controlling for the a set of 53 annotations^44^, including standard genomic annotations such as exon, intron, 3′UTR, 5′UTR, presence of enhancers, total LD score, and brain gene affiliation. Given the complex LD^75^ in the extended major histocompatibility complex (MHC) region (genome build 19 location 25119106–33854733), we excluded SNPs in the MHC region and SNPs in LD (r^2^ > 0.1) with such SNPs from the analysis, to avoid any inflation due to complex correlations. We then used the LD-score tool with the “—l2” option to calculate the total LD of 1,190,321 variants from the HapMap3 project towards the category of variants with negative scores. The pairwise LD r^2^ measures were calculated across 9,997,231 variants from the reference panel (1 kG Phase3 genotypes for individuals with European descent). The effect reported (β_PNSS_) is the LD score regression coefficient. Its p-value is the probability of the “true” effect size being different from zero based on the standard error estimate of the coefficient.

### Data can be obtained from

EduYears: https://www.thessgac.org/data, College: http://www.ccace.ed.ac.uk/node/335, GCA1:https://ctg.cncr.nl/software/summary_statistics, GCA 2: data can be obtained from the CHARGE consortium, BMI and Height:, http://portals.broadinstitute.org/collaboration/giant/index.php/GIANT_consortium_data_files.

## Electronic supplementary material

Supplementary Information
