# Supplementary material for: Enrichment of genetic markers of recent human evolution in educational and cognitive traits
Source: Sci Rep. 2018 Aug 22;8:12585. doi: 10.1038/s41598-018-30387-9 (PMC6105609; doi:10.1038/s41598-018-30387-9)
Supplement: Supplementary file 1 — Supplementary Information [file 41598_2018_30387_MOESM1_ESM.pdf]

Enrichment of genetic markers of recent human evolution in educational and cognitive traits.

Saurabh Srinivasan [1,2], Francesco Bettella [1,2], Oleksandr Frei [1,2], W. David Hill [3,4], Yunpeng Wang [1,2], Aree Witoelar [1,2], Andrew J. Schork [8], Wesley K. Thompson [7,8], Gail Davies [3,4], Rahul S. Desikan [9], Ian J. Deary [3,4], Ingrid Melle [1,2], Torill Ueland [1,2], Anders M. Dale [5,6,10,10], Srdjan Djurovic [11,12], Olav B. Smeland [1,2], Ole A. Andreassen [1,2*]

1 NORMENT, KG Jebsen Centre for Psychosis Research, Institute of Clinical Medicine, University of Oslo, Oslo, Norway

2 Division of Mental Health and Addiction, Oslo University Hospital, Oslo, Norway

3 Centre for Cognitive Ageing and Cognitive Epidemiology, University of Edinburgh, Edinburgh, UK

4 Department of Psychology, University of Edinburgh, Edinburgh, UK

5 Multimodal Imaging Laboratory, University of California at San Diego, La Jolla, CA, USA

6 Center for Human Development, University of California at San Diego, La Jolla, CA, USA

7 Institute of Biological Psychiatry, Mental Health Center St. Hans, Mental Health Services Copenhagen, Roskilde, Denmark.

8 Department of Family Medicine and Public Health, University of California, San Diego, La Jolla, CA, USA

9 Neuroradiology Section, Department of Radiology and Biomedical Imaging, University of California at San Francisco, San Francisco, CA, USA

10 Department of Psychiatry, University of California, San Diego, La Jolla, CA, USA

11 Department of Medical Genetics, Oslo University Hospital, Oslo, Norway

12NORMENT, KG Jebsen Centre for Psychosis Research, Department of Clinical Science, University of Bergen, Bergen, Norway

*Corresponding author

Email: o.a.andreassen@medisin.uio.no

Supplementary Material

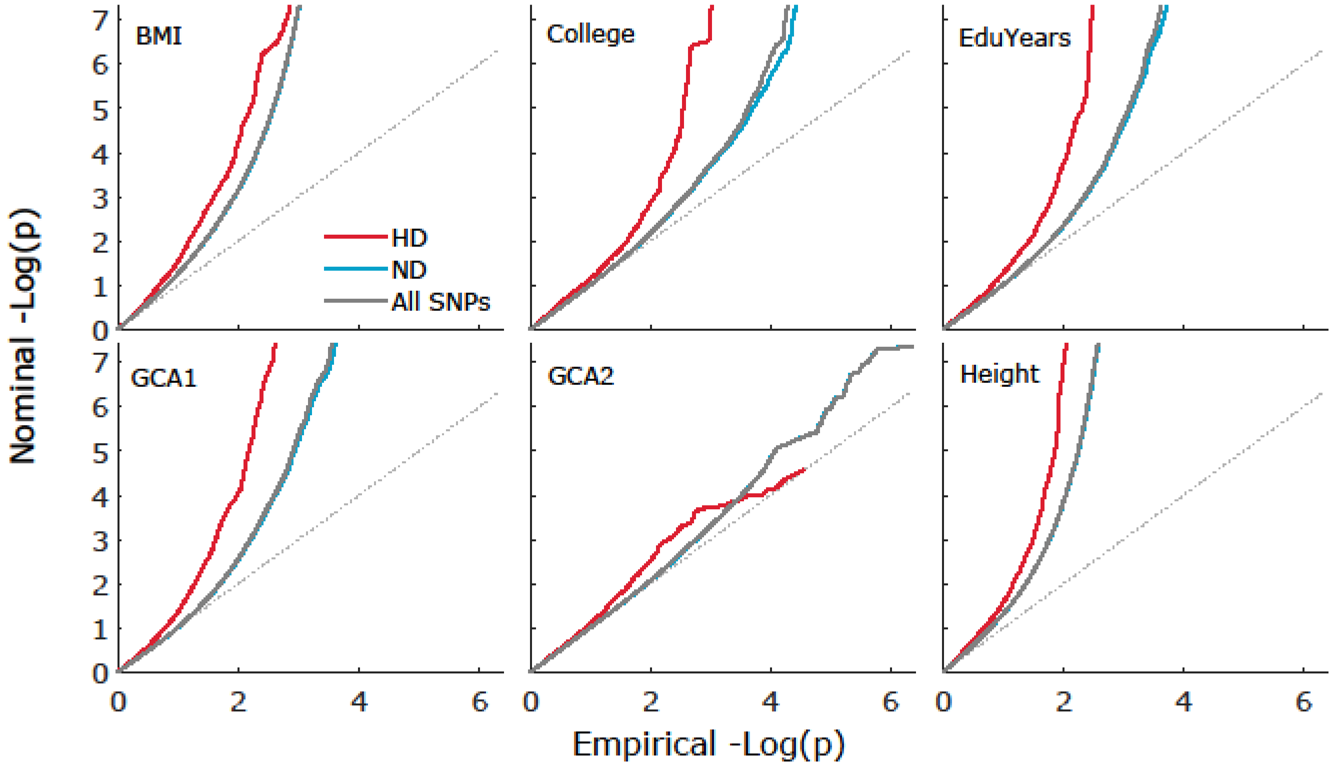

Supplementary Fig. S1: Enrichment of associations with various traits stratified according to the Post-Neanderthal selective sweep region affiliation. Conditional Q-Q plots of GWAS summary statistics *p*-values for body mass index (BMI), college completion (College), educational attainment (EduYears), general cognitive ability (GCA1 and 2) and height, stratified based on the Post-Neanderthal selective sweep (PNSS) region affiliation. The human divergent (HD) stratum comprises genetic variants in swept regions. The non-divergent (ND) stratum comprises genetic variants outside the swept regions. HD variants show some leftward deflection from ND and all SNPs. This signifies a comparatively higher proportion of low p-values among HD variants

	Enrichment	
**Phenotype**	**β_{PNSS}**	**p-value**
**BMI**	5.58×10^{-9}	2.52×10^{-1}
**College**	2.75×10^{-8}	$1.95\times10^{-2*}$
**EduYears**	1.56×10^{-8}	$4.58\times10^{-2*}$
**GCA1**	2.72×10^{-8}	$2.89\times10^{-2*}$
**GCA2**	4.63×10^{-8}	$2.01\times10^{-2*}$
**Height**	4.97×10^{-8}	8.00×10^{-2}

* Nominally significant

Supplementary table S1. **Stratified enrichment analysis for genetic variants in regions that possibly underwent a selective sweep after divergence from Neanderthal**. Cognitive measures: college completion (College), educational attainment (EduYears), general cognitive ability (GCA1 and 2); Anthropometric measures: body mass index (BMI) and Height. The table shows the LD score regression model enrichment test statistics: the PNSS affiliation score ANCOVA coefficient (β_{PNSS}) and the corresponding p-values.

Phenotype	Enrichment					
	Brain		HD		HDBrain	
	Fold	p-value	Fold	p-value	Fold	p-value
**BMI**	1.10	5.03E-02	1.38	3.12E-01	2.58	1.13E-01
**College**	1.08	3.00E-01	2.33	2.63E-02*	2.16	3.42E-01
**EduYears**	1.07	1.42E-01	2.00	4.48E-02*	1.83	3.71E-01
**GCA1**	1.09	3.27E-02*	2.12	2.45E-02*	1.68	3.77E-01
**GCA2**	1.24	3.60E-02*	3.48	2.36E-02*	4.75	3.65E-02*
**Height**	1.21	2.03E-06*	2.10	7.17E-02	1.99	2.30E-01

* Nominally significant

Supplementary table S2. **Stratified Enrichment of associations with various traits stratified according to their affiliation to Post-Neanderthal selective sweep regions and brain genes** Cognitive measures: college completion (College), educational attainment (EduYears), general cognitive ability (GCA1 and 2); Anthropometric measures: body mass index (BMI) and Height. The table shows the LD score regression model enrichment test statistics: the enrichment of associations (Fold) among variants in the human divergent regions (HD) compared to variants expressed in the brain (Brain) and the brain genes found in the human divergent regions (HDBrain) other variants and the corresponding p-values.
